# Proteomics and weighted gene correlated network analysis reveal glutamatergic synapse signaling in diazepam treatment of alcohol withdrawal

**DOI:** 10.3389/fphar.2022.1111758

**Published:** 2023-01-11

**Authors:** Wan Kong, Shanqing Huang, Zikai Chen, Xiaolin Li, Shujing Liu, Zi Zhang, Ye Yang, Zhanzhang Wang, Xiuqing Zhu, Xiaojia Ni, Haoyang Lu, Ming Zhang, Zezhi Li, Yuguan Wen, Dewei Shang

**Affiliations:** ^1^ Department of Pharmacy, The Affiliated Brain Hospital of Guangzhou Medical University, Guangzhou, China; ^2^ Department of Administration, The Affiliated Brain Hospital of Guangzhou Medical University, Guangzhou, China; ^3^ Department of Adult Psychiatry, The Affiliated Brain Hospital of Guangzhou Medical University, Guangzhou, China

**Keywords:** alcohol withdrawal, diazepam, glutamatergic synapse, proteome, weighted gene correlated network analysis

## Abstract

**Background:** Alcohol use disorder (AUD) is characterized by chronic excessive alcohol consumption, often alternating with periods of abstinence known as alcohol withdrawal syndrome (AWS). Diazepam is the preferred benzodiazepine for treatment of alcohol withdrawal syndrome under most circumstances, but the specific mechanism underlying the treatment needs further research.

**Methods:** We constructed an animal model of two-bottle choices and chronic intermittent ethanol exposure. LC-MS/MS proteomic analysis based on the label-free and intensity-based quantification approach was used to detect the protein profile of the whole brain. Weighted gene correlated network analysis was applied for scale-free network topology analysis. We established a protein–protein interaction network based on the Search Tool for the Retrieval of Interacting Genes (STRING) database and Cytoscape software and identified hub proteins by CytoHubba and MCODE plugins of Cytoscape. The online tool Targetscan identified miRNA–mRNA pair interactions.

**Results:** Seven hub proteins (Dlg3, Dlg4, Shank3, Grin2b, Camk2b, Camk2a and Syngap1) were implicated in alcohol withdrawal syndrome or diazepam treatment. In enrichment analysis, glutamatergic synapses were considered the most important pathway related to alcohol use disorder. Decreased glutamatergic synapses were observed in the late stage of withdrawal, as a protective mechanism that attenuated withdrawal-induced excitotoxicity. Diazepam treatment during withdrawal increased glutamatergic synapses, alleviating withdrawal-induced synapse inhibition.

**Conclusion:** Glutamatergic synapses are considered the most important pathway related to alcohol use disorder that may be a potential molecular target for new interventional strategies.

## Graphical Abstract

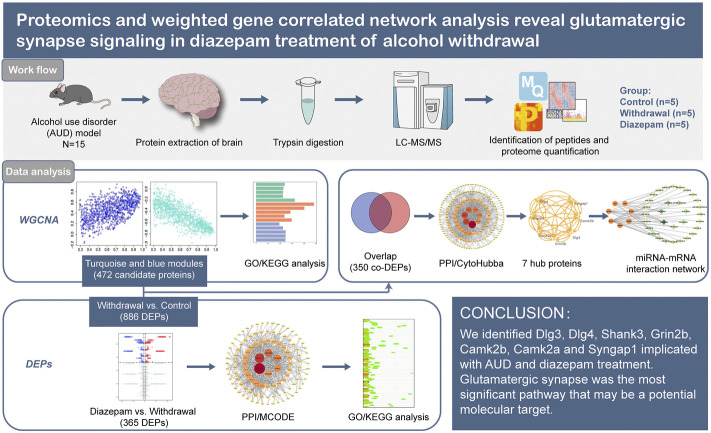

## 1 Introduction

Alcohol use disorder (AUD) is characterized by chronic excessive alcohol consumption, often alternating with periods of abstinence accompanied by symptoms of tremors, anxiety, irritability, and agitation, collectively known as alcohol withdrawal syndrome (AWS). AUD is associated with neurological deficits including loss of brain volume and cognitive impairments (Sullivan et al., 2000). Emerging evidence indicates that dysfunctional glutamate neurotransmission is critical in the initiation and development of alcohol and drug dependence (Goodwani et al., 2017). Our research showed that Glutamatergic synapses may be a potential molecular target for new interventional strategies.

Transcriptional analysis is a useful method for determining changes in gene expression; however, the results do not always accurately correlate with protein levels. Therefore, application of proteomic analysis to animal models of AUD is necessary to provide a new understanding of mechanisms underlying associated neuroplasticity, and to identify new therapeutic targets for AUD. Weighted gene correlated network analysis (WGCNA) converts gene expression data into networks, or modules, containing a group of genes that share a common biological relationship or function and behave similarly, thereby providing modules that may be responsible for the phenotypic characteristics of interest. WGCNA has been applied to the study of alcohol dependence (Nunez et al., 2013; Mamdani et al., 2015; Kapoor et al., 2019).

Diazepam is the preferred benzodiazepine for treatment of patients experiencing moderate to severe AWS (Weintraub, 2017), but the specific mechanism underlying the effects of treatment needs further research. Here, we used LC-MS/MS proteomic analysis based on the label-free and intensity-based quantification (iBAQ) approach in animal models of diazepam treatment of AUD. We used WGCNA scale-free network algorithms combined with bioinformatic methods to analyze chronic intermittent ethanol exposure simultaneously with diazepam treatment-evoked changes in protein levels in brain tissue. We established a protein–protein interaction (PPI) network based on the Search Tool for the Retrieval of Interacting Genes (STRING) database and Cytoscape software to identify hub genes related to AUD. Subsequently, the hub gene and miRNA–mRNA pair interactions were identified. We screened the hub genes and pathways highly associated with AUD and provided further insight into the pathophysiology of AUD at the molecular level and explored potential molecular targets for new interventional strategies.

## 2 Material and methods

### 2.1 Animals

Adult male C57BL/6J mice purchased from Guangdong Provincial Medical Laboratory Animal Center were individually housed in an animal facility with corncob bedding under a 12-h light–dark cycle. The temperature was kept constant at 24°C ± 2°C, and relative humidity was maintained at 60 ± 5%. Mice were given free access to food and tap water during all experimental procedures. All animal procedures were approved by the Experimental Animal Ethics Committee of Guangdong Medical Experimental Animal Center (permit number: C202207-26). In the animal experiments, all operations and treatments are obliged to conform to the Declaration of Helsinki and the “3R” principles.

### 2.2 Ethanol consumption

After a 2-week adaptation period (age 7-8 weeks), the experimental mice were weighed. The two-bottle choice was given as described previously (Juarez et al., 2017). Ethanol concentration successively increased from 3% (v/v solution) to 6% and 10%, and each concentration was administered for 4 days. Bottles were weighed every 2 days and interchanged to prevent side preference. Mice were weighed every 4 days (i.e., once for each concentration of ethanol). On the fourth day of access to 10% ethanol and water, individual drinking behaviors were determined. Ethanol preference (%) was determined as ethanol intake/total fluid intake × 100%. Ethanol consumption (g/kg/day) was determined as ethanol intake × ethanol concentration/mouse weight per day. Low alcohol consumption mice had an ethanol preference <40% and consumption <10 g/kg/day. High alcohol consumption mice had an ethanol preference >70% and consumption >10 g/kg/day. Mice that did not meet these criteria (medium alcohol consumption) were not used.

After two-bottle choices, mice were subjected to chronic intermittent ethanol exposure. One bottle provided 20% alcohol for 7 days followed by alcohol deprivation for 2 days (abstinence period), and then 35% alcohol for 7 days, corresponding to the reinstatement stage. During the second abstinence period, diazepam (Shanghai Xudong Haipu, China) treatment was initiated at 2.6 mg/kg (bid) by intraperitoneal injection. The dosing volume was .1 mL/10 g. The mice were sacrificed by intraperitoneal injection of excessive Pentobarbital sodium followed by the brain tissue samples were extracted on ice and stored in −80°C.

### 2.3 Protein extraction and trypsin digestion

Samples were minced and lysed in lysis buffer (Thermo Fisher Scientific, Rockford, IL, United States) containing protease inhibitors, and phosphatase inhibitors (Thermo Fisher Scientific, Rockford, IL, United States) followed by 3 min of heat at 95°C and 5 min of sonication on ice after cooling to room temperature. The lysate was centrifuged at 14,000×*g* for 10 min and the supernatant was collected as whole tissue extract. Bradford protein assay was used to determine protein concentration. Ammonium bicarbonate solution (50 mM) was added to 30 μg protein extracts from each sample for enzymatic digestion. The digested peptides were lyophilized, desalted, re-lyophilized, and redissolved in 12 μL .1% formic acid solvent, and quantified.

### 2.4 LC-MS/MS

Samples were separated on Easy-nLC 1,000 nanoflow LC system (Thermo Fisher Scientific). Solvent A was .1% formic acid in water, and Solvent B was .1% formic acid in 80% acetonitrile solution. After equilibrating the column with 100% Solvent A, the peptide samples were loaded onto the sample column by the autosampler and separated by the analytical column at a flow rate of 600 nL/min for 75 min.

MS was performed using a Q Exactive HF-X mass spectrometer (Thermo Fisher Scientific) with one full scan (300–1,400 m/z, R = 120,000 at 200 m/z, positive ion mode) at automatic gain control target of 3e6 ions with a maximum injection time of 80 ms. Dynamic exclusion time was set at 40.0 s. After each full scan, the most intense ions selected under top-speed mode were isolated with a 1.6 m/z window and fragmented by higher-energy collisional dissociation with a normalized collision energy of 27%. The 60 fragment spectra were collected by MS/MS scans with a resolution of 7,500 at 200 m/z. The Q Exactive HF-X mass spectrometer with high accuracy and high resolution guaranteed obtaining high-quality MS1 and MS2 spectra.

### 2.5 Data pretreatment and identification of differentially expressed proteins

The raw MS data were in RAW files, and Firmiana cloud platform was used for database identification and quantitative analysis, including missing value imputation, log2 transformed background adjustment, quantile normalization, and principal components analysis (PCA) (Feng et al., 2017). If the samples did not conform to a normal distribution, a Wilcoxon rank-sum test was used to identify proteins with significantly different expression in the control group *versus* the withdrawal group and the withdrawal group *versus* the diazepam group. *p* < .05 and |log2FC| > 1 were considered indicative of significant differences in protein expression between the two groups. The volcano plot and hierarchical clustering heatmap were generated using R software 4.2.0 (https://www.r-project.org). The Venn diagram was constructed using online website (https://bioinformatics.psb.ugent.be/webtools/Venn/).

### 2.6 WGCNA

WGCNA performed with the WGCNA package (version 1.6.9) for R software 4.2.0 (https://www.r-project.org) was used for scale-free network topology analysis (Langfelder and Horvath, 2008). WGCNA clustered genes with similar expression patterns into modules and showed the relation between modules and specific traits. The process mainly included the following steps: 1) defining the similarity matrix and transforming it into the adjacencies matrix according to the weight coefficient β selected; 2) transforming the adjacencies matrix into the topological overlap matrix (TOM); 3) the hierarchical clustering tree was obtained by the hierarchical clustering of TOM-based dissTOM; 4) dynamic tree cutting method was used to identify modules from hierarchical clustering tree; and 5) calculating the eigengene for each module (ME) (Xu et al., 2022; Zhao et al., 2022; Zhong et al., 2022). ME represented the overall expression level of the module. Pearson correlation coefficient between MEs of each module was calculated (Guillotin et al., 2021). Standard WGCNA parameters were used for analysis, with the exceptions of soft-thresholding power and deep split. A soft-thresholding power of 12 was used, which was selected using methods described by Langfelder (Langfelder et al., 2008). A deep split value of three was selected and the minimum number of genes per module was defined as 30.

The correlation between modules and traits was described with a heatmap to identify the modules most closely implicated with traits. A |correlation coefficient| > .5 and *p* < .05 were cut-offs for module screening. For each module, module membership (MM) was the correlation between a given gene expression profile and ME of a given model. Gene significance (GS) was defined as the value of the correlation between a gene and a trait. The ME in the key modules with MM > .8 and |GS| > .2 were selected for further analysis.

### 2.7 Gene ontology and KEGG pathway analysis

To examine for potential biological process (BP), molecular function (MF), cellular component (CC) and related pathways of turquoise and blue modules, Gene Ontology (GO) analysis (https://www.geneontology.org/) and the Kyoto Encyclopedia of Genes and Genomes (KEGG) pathway analysis (https://www.genome.jp/kegg/pathway.html) were performed by DAVID 2021 (https://david.ncifcrf.gov). *p* < .05 was considered to indicate a statistically significant difference.

### 2.8 Bioinformatic analysis

Overlap was determined between genes in the turquoise module verified by WGCNA and differentially expressed proteins (DEPs; in withdrawal compared to control group) using online veen tools (https://www.vandepeerlab.org). The online search tool STRING database (STRING, V11.5; https://cn.string-db.orgHYPERLINK https://cn.string-db.org) was applied to predict PPIs, including functional associations and physical interactions (Szklarczyk et al., 2021). The PPI pairs with a combined score ≥.7 were considered significant and outlier proteins were removed. Cytoscape software (https://cytoscape.org, version 3.9.1) was used to construct and visualize the PPI and miRNA–mRNA interaction networks. To further determine hub proteins in BP, CytoHubba (version .1) plugins of Cytoscape was applied to measure the interaction of candidate proteins based on four algorithms: edge percolated component (EPC), maximal clique centrality (MCC), degree, the maximum neighborhood component (MNC). A Venn diagram was drawn to show the overlapping proteins. The molecular complex detection (MCODE) algorithm, a plugin in Cytoscape, was used for clustering a given network based on topology to find densely connected regions (Bader and Hogue, 2003). The advanced options set as degree cutoff = 2, K-core = 2, and node score cutoff = .2. The online tool Targetscan (Release 7.2; https://www.targetscan.org/mmu_72/) was used to identify miRNAs that may regulate hub mRNA and miRNAs (Agarwal et al., 2015). The miRNA with total context++ score ≤ −.2 and conserved in Mammals were selected. We used Cytoscape software to construct interaction networks of mRNA–miRNA pairs with inverse expression associations.

## 3 Results

### 3.1 Low or high alcohol consumption groups were determined by two-bottle choice

**FIGURE 1:**
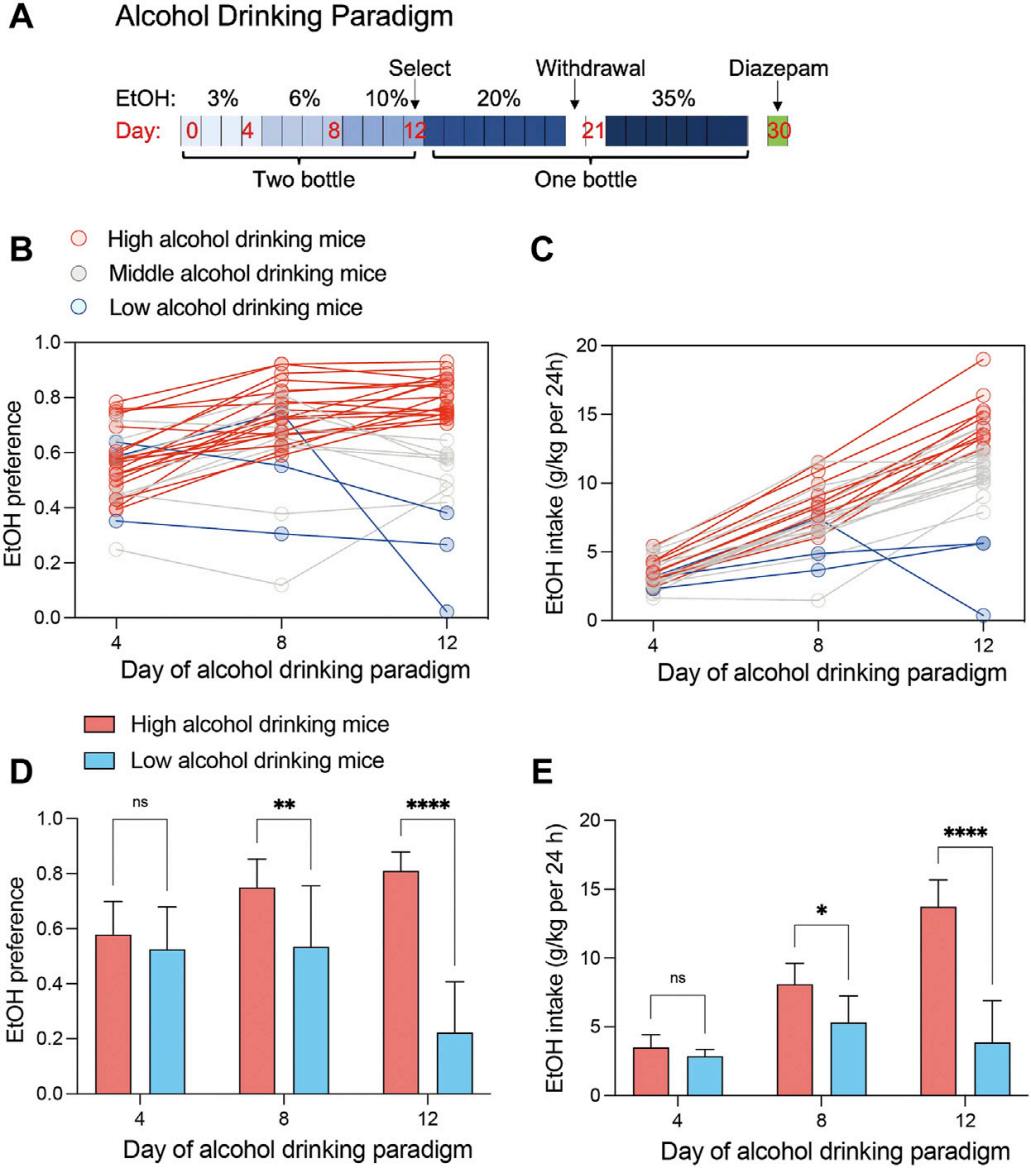
Alcohol consumption of C57BL/6J mice (n = 30). **(A)** A 12-day continuous-access two-bottle choice of alcohol consumption was performed, followed by determination of alcohol consumption behavior. Mice continued to be supplied water or ethanol in one bottle and deprived of ethanol twice (2 days). **(B)** Individual ethanol preference scatters map across day of alcohol drinking procedure. Data show preference for 3% ethanol (day 4), 6% ethanol (day 8), and 10% ethanol (day 12). **(C)** Individual ethanol intake scatters map across day of alcohol drinking procedure. Data show daily intake of 3% ethanol (day 4), 6% ethanol (day 8), and 10% ethanol (day 12). **(D)** Ethanol preferences in high (n = 19) and low (*n* = 3) alcohol consumption groups (two-way ANOVA: interaction effect F (2, 60) = 15.64, *p* < .0001; alcohol consumption group effect F (1, 60) = 50.88, *p* < .0001; Šídák’s multiple comparisons test, **p < .01, ****p < .0001). **(E)** Ethanol intake in high (*n* = 19) and low (*n* = 3) alcohol consumption groups (two-way ANOVA: interaction effect F (2, 60) = 23.82, *p* < .0001; drinking group effect F (1, 60) = 60.04, *p* < .0001; Šídák’s multiple comparisons test, *p < .05, ****p < .0001).

Male C57BL/6J mice aged 7-8 weeks were allowed to voluntarily consume water and increasing concentrations of alcohol (4 days each concentration: 3%, 6% and 10% v/v ethanol) over 12 days (Figure 1A). Alcohol consumption behavior changed on day 8, with further changes on day 12 (Figures 1B, C). The results suggested that EtOH preference can sufficiently distinguish mice because of EtOH preferences of 30 mice ranged from .93 to .02 on day 12 (Figure 1B). On day 12, 63.3% of mice showed >70% alcohol preference and 10% showed <40%. Alcohol intake increased with concentration, among which, 83.3% of the mice consumed >10 g/kg/day and 16.7% consumed <10 g/kg/day (Figure 1C). Thus, we considered mice with <40% alcohol preference and <10 g/kg/day alcohol intake to have low alcohol consumption (*n* = 3), and mice with >70% alcohol preference and >10 g/kg/day alcohol intake to have high alcohol consumption (*n* = 19). The classification was proved reasonable by less EtOH preference and EtOH intake of Low alcohol drinking mice than high alcohol drinking mice, especially at 8 and 12 days (Figures 1D, E).

### 3.2 Chronic intermittent ethanol exposure and withdrawal

After two-bottle choice, the mice were divided into three groups by alcohol preference and chronic intermittent ethanol exposure and withdrawal were implemented. The five mice with the lowest alcohol preference were considered as the control group (*n* = 5). The ten mice with the highest alcohol preference were divided into the withdrawal group (*n* = 5) and diazepam group (*n* = 5). The control group was provided with water until death. The withdrawal group was provided with alcohol and withdrawn twice. The diazepam group was the same as the withdrawal group except for receiving diazepam on the second day of withdrawal.

### 3.3 Data processing and identification of DEPs

LC-MS/MS measured 15 samples and demonstrated good consistency in proteome identification and quantification. The mass deviation of all identified peptide segments was mainly distributed within 10 ppm, indicating accurate and reliable results (Supplementary Figure S1A). The number of proteins identified in each sample was highly consistent; however, each group contained 5%–12% specific proteins (Supplementary Figures S1B, D). The cumulative number of proteins was 6,536, achieving deep coverage of the proteome (Supplementary Figure S1C, Table 1). Proteome quantification was performed by the iBAQ algorithm followed by normalization to the fraction of total (Supplementary Figures S2A–C). To evaluate the sample differences within each group (intra-group deviation), correlation analysis was performed. The results showed that the correlation of the samples in each group was high (.81–.99), suggesting good experimental repeatability (Supplementary Figure S2D).

**TABLE 1:** Number of spectra, identified peptide segments and proteins by each sample.

Id	Spectrum	Peptides	Protein
Exp115928_C10	1,29,018	24,596	4,689
Exp115929_C38	1,26,194	23,864	4,605
Exp115930_C21	1,27,774	24,273	4,678
Exp115931_C1	1,29,337	25,655	4,860
Exp115932_C16	1,25,989	23,143	4,572
Exp115933_W7	1,28,839	22,713	4,716
Exp115934_W39	1,29,543	24,064	4,710
Exp115935_W23	1,27,226	23,447	4,668
Exp115936_W31	1,28,820	25,101	4,878
Exp115937_W26	1,28,328	23,855	4,695
Exp115938_D34	1,34,571	28,059	5,165
Exp115939_D9	1,29,702	23,980	4,729
Exp115940_D29	1,29,592	25,112	4,860
Exp115941_D32	1,32,147	25,485	4,816
Exp115942_D40	1,33,149	25,447	4,809

**FIGURE 2:**
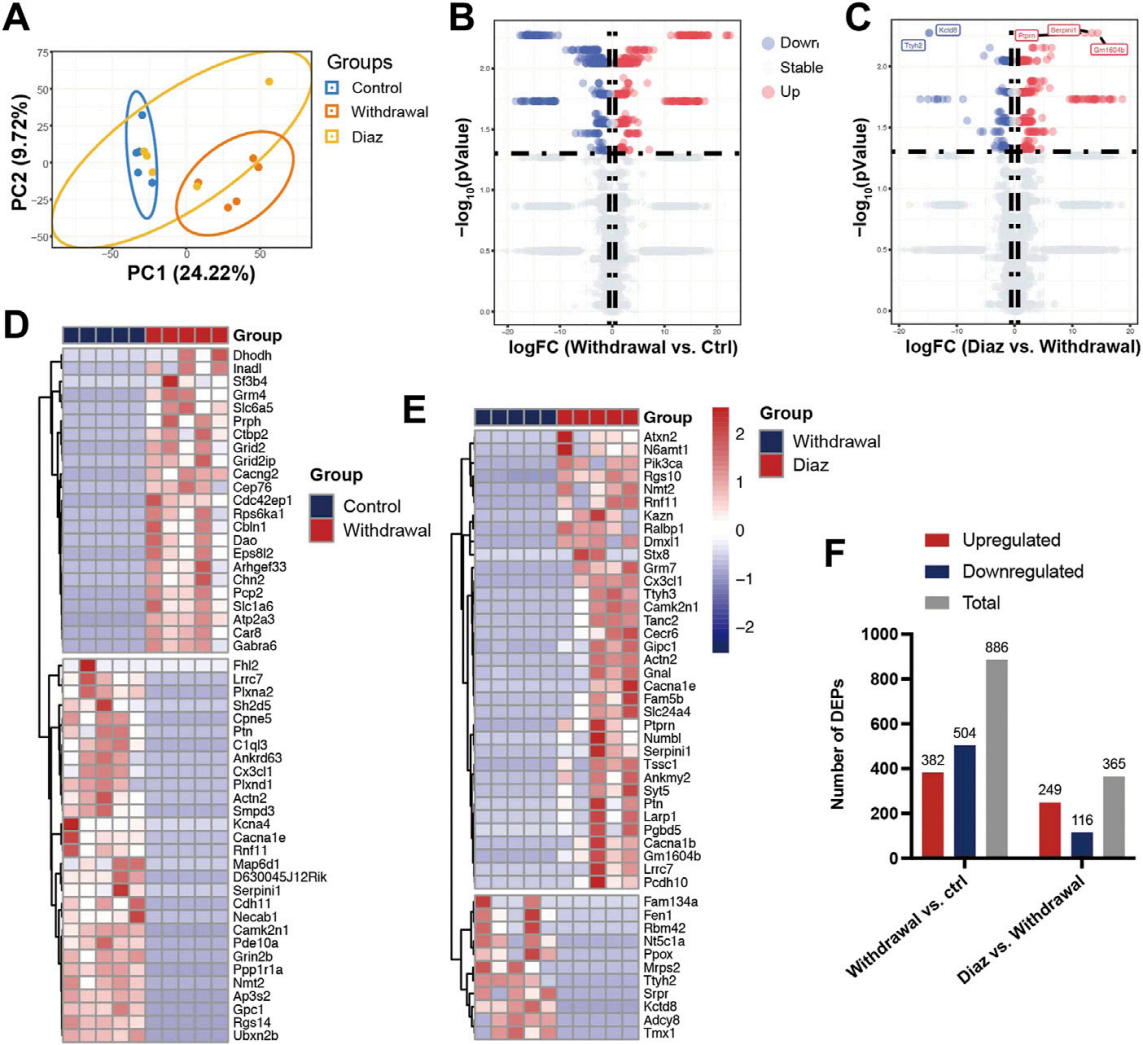
Identification of DEPs. **(A)** PCA plots of Control (blue), Withdrawal (orange) and Diazepam (yellow) Groups in 15 samples with oval confidence intervals. **(B)** Volcano plot of Control and Withdrawal Group samples. **(C)** Volcano plot of Withdrawal and Diazepam Group samples. Colors represent different genes: grey nodes represent proteins without significantly different expression, red nodes represent upregulated proteins, and blue nodes represent downregulated proteins. **(D)** Hierarchical clustering heatmap of 52 DEPs (represented by rows) in Control and Withdrawal Group samples (represented by columns) and **(E)** 46 DEPs in Withdrawal and Diazepam Group samples. Colors represent relative abundance of proteins using normalized intensity data. **(F)** Number of DEPs in Withdrawal *versus* Control and Diazepam *versus* Withdrawal Group samples. Red bar represents upregulated DEPs. Blue represents downregulated DEPs. Grey represents total DEPs.

PCA, an unsupervised data analysis method, revealed the overall distribution trend of samples between groups, showing that the samples between the control group and withdrawal group were scattered well and the samples within the group were well clustered together. However, the diazepam group could not be distinguished from the other two groups, probably resulting from the over-short treatment time of diazepam (1 day) (Figure 2A). Compared with the control group, we identified 886 DEPs (382 upregulated and 504 downregulated) in the withdrawal group and 365 (249 upregulated and 116 downregulated) in the diazepam group compared with withdrawal group (Figure 2F). All proteins are shown in the volcano plot (Figures 2B, C). The most significant DEPs were shown in the hierarchical clustering heatmap (Figures 2D, E). In the withdrawal and control groups, Ppp1r1a was the most significant DEP (logFC = −16.02, *p* = .005) with protein serine/threonine phosphatase inhibitor activity, involved in intracellular signal transduction (Cataldo et al., 2021). However, in the diazepam and withdrawal groups, Serpini1 was the most significant DEP (logFC = 13.36, *p* = .005), playing a role in the regulation of axonal growth and development of synaptic plasticity (Hermann et al., 2020).

### 3.4 WGCNA

**FIGURE 3:**
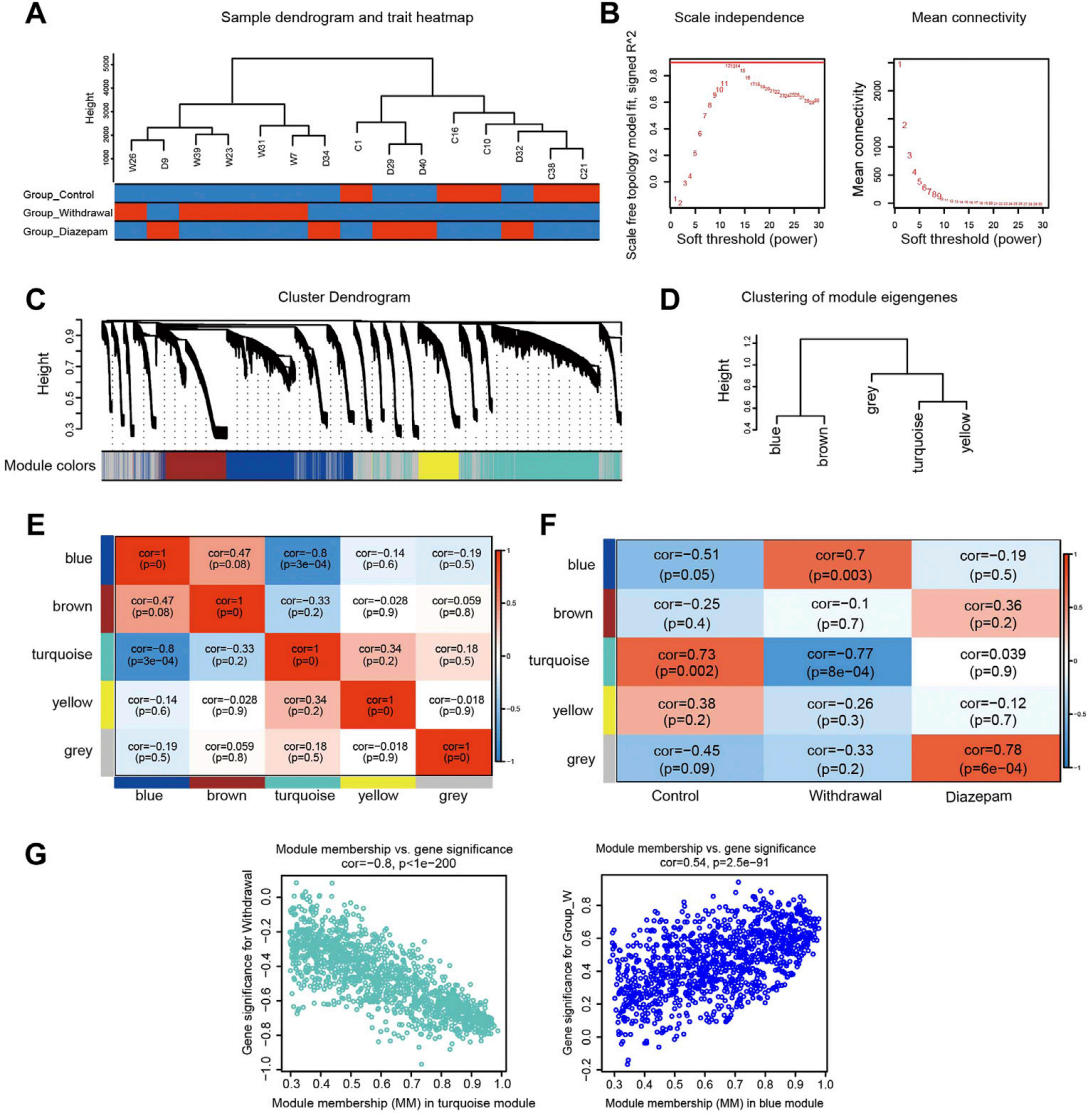
WGCNA identified modules associated with alcohol withdrawal. **(A)** Sample cluster dendrogram detected outliers and the trait heatmap displayed the sample traits. **(B)** Soft-thresholding power selection of WGCNA. The left panel shows the scale-free fit index as a function of the soft-thresholding power. The right panel shows the mean connectivity as a function of soft-thresholding powers. **(C)** Module assignment and cluster dendrogram. Highly interconnected genes were clustered and five modules were identified with hierarchical clustering tree analysis. Different colors represent different modules. **(D)** Cluster dendrogram of five modules. **(E)** Heatmap of the correlations among modules based on Pearson’s correlation coefficient. Correlation of the corresponding modules is represented by square colors. High correlation is represented with red, while low correlation is represented with blue. **(F)** The module–trait relationship heatmap. The columns represent Control, Withdrawal, and Diazepam Groups. The rows represent the module eigengenes. Corresponding correlations and *p* values are shown in each square. Blue represents a negative correlation, and red a positive correlation. **(G)** Scatterplot of genes in the turquoise and blue modules; the correlation and *p*-value are under the title.

Compared with DEP analysis, WGCNA constructed the scale-free network and was therefore of more biological significance. A sample cluster dendrogram showed that no outliers were observed, hence, all samples were used for analysis (Figure 3A). Choosing soft-thresholding power = 12 to construct the expression network was reasonable according to Figure 3B. A hierarchical clustering tree was obtained by conducting hierarchical clustering for dissTOM and five modules were identified: blue, brown, grey, turquoise and yellow (grey module was the no significant module, with no follow-up analysis; Figures 3C, D). The heatmap quantified module similarity through eigengene correlation. The results indicated that the blue and turquoise modules had the lowest similarity (correlation = −.8; Figure 3E), suggesting the proteins in the two modules had the opposite expression pattern. The associations between traits and modules were identified according to the correlation between module eigengene and traits (Figure 3F). The two modules were significantly correlated with clinical characteristics of withdrawal. The blue module (1,194 proteins) was positively related to withdrawal, and the turquoise module (1,386 proteins) was negatively correlated. They included most of the proteins that were over- or underexpressed in withdrawal, respectively. Therefore, the blue and turquoise modules were treated as withdrawal-related modules in subsequent analyses (Figure 3G). However, the modules closely related to diazepam treatment were not discovered, which may have resulted from the over-short treatment time for diazepam. We used *p* < .05, |GS| > .2 and MM > .8 to screen key proteins in the blue and turquoise modules and obtained 472 candidate proteins that were highly correlated with withdrawal, which were included for further analysis.

### 3.5 GO functional and KEGG pathway enrichment analysis of turquoise and blue modules

**FIGURE 4:**
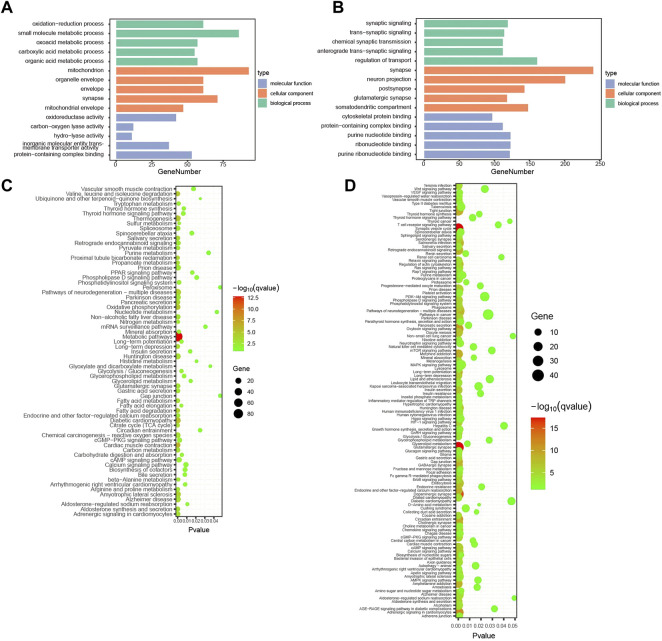
Enrichment analysis of withdrawal-related turquoise and blue modules. **(A)** Top 15 GO functional annotations in blue module. **(B)** Top 15 GO functional annotations in turquoise module. **(C)** KEGG pathway enrichment analysis in blue module. **(D)** KEGG pathway enrichment analysis in turquoise module.

To further investigate the functions and mechanisms of the turquoise and blue modules negatively and positively related to the alcohol withdrawal respectively, GO and KEGG pathway enrichment analyses were performed. The enriched GO annotations of the blue module included small molecule metabolic process in the BP category, mitochondrion in the CC category, and protein-containing complex binding in the MF category (Figure 4A). KEGG pathways mainly included metabolic pathways (GeneRatio = 86/206, p.adjust = 2.21 × 10^–13^; Figure 4C). The above results suggested that alcohol withdrawal was implicated with small molecule metabolic pathways, consistent with many previous reports; for example: β-hydroxybutyrate metabolism linked to AUD (Leclercq et al., 2020); kynurenine metabolism impairing alcohol seeking and relapse (Vengeliene et al., 2016); tryptophan metabolism associated with alcohol dependence (Zhu et al., 2021); and glutathione and lipid peroxidation (Videla and Valenzuela, 1982). For the turquoise module, the enriched GO annotations included regulation of transport in the BP category, synapse in the CC category, and ribonucleotide binding in the MF category (Figure 4B). KEGG pathways mainly included glutamatergic synapse (generation = 32/330, q = 4.18 × 10^–18^), synaptic vesicle cycle (generation = 27/330, q = 4.18 × 10^–18^), dopaminergic synapse (generation = 32/330, q = 4.96 × 10^–16^), calcium signaling pathway (generation = 28/330, q = 3.43 × 10^–7^) (Figure 4D). The results indicated that the above pathways were downregulated in alcohol withdrawal mice. For more reliable analysis, we combined WGCNA with differentially expressed analysis to carry out overlap analysis and identify hub proteins.

### 3.6 Identification of hub proteins and construction of miRNA–mRNA interaction network

**FIGURE 5:**
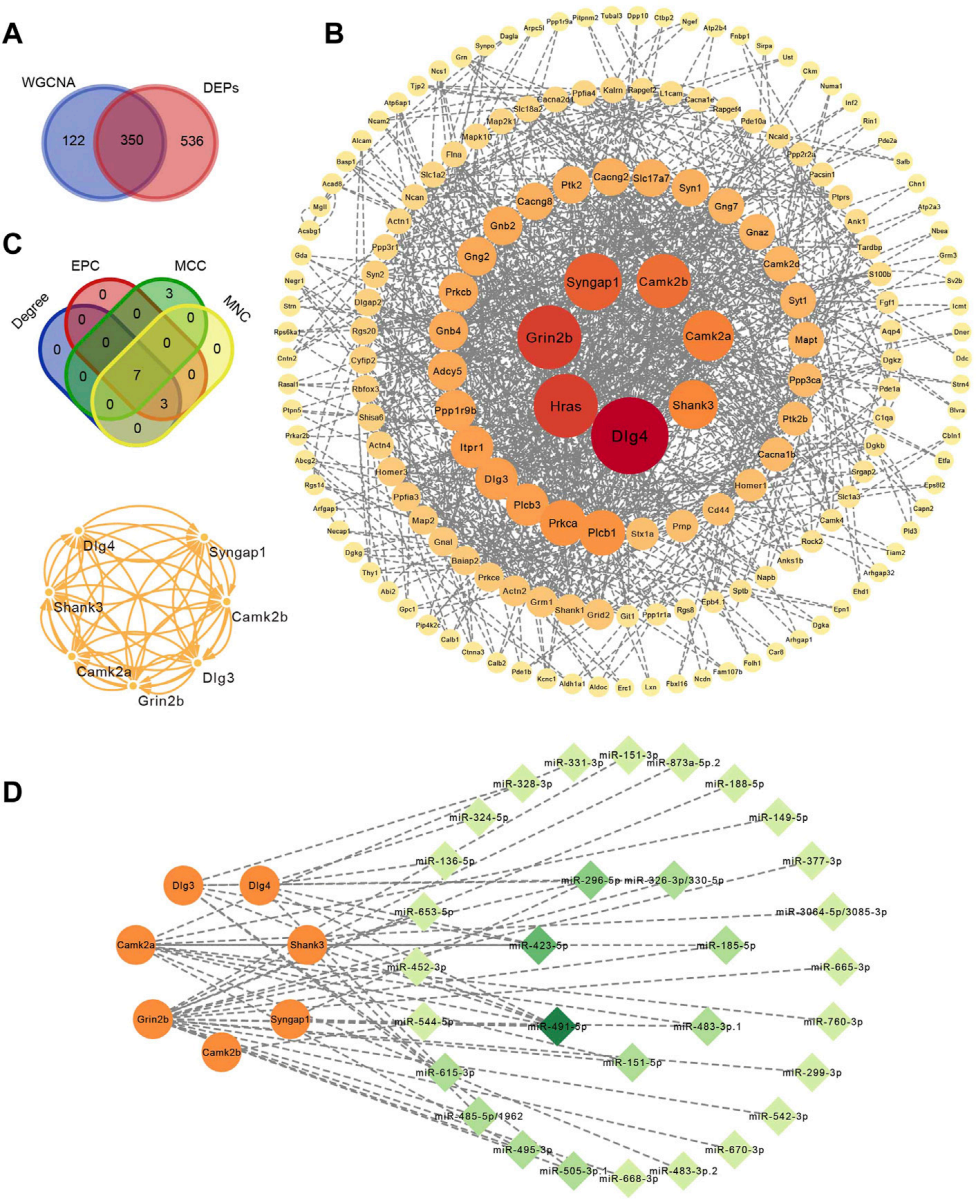
Identification of hub proteins related to alcohol withdrawal. **(A)** Overlap analysis in 472 proteins in turquoise module (MM > .8 and |GS| > .2) verified by WGCNA and 886 DEPs. **(B)** PPI network construction of 350 overlapped proteins related to alcohol withdrawal. **(C)** Seven hub proteins related to withdrawal identified by CytoHubba plugins of Cytoscape based on four algorithms: EPC, MCC and MNC. **(D)** miRNA–mRNA interactions network. Orange circle represents hub proteins and green diamond miRNA. The deeper the color and the larger the circle, the greater the degree.

Four hundred and seventy-two proteins in the turquoise module (MM > .8 and |GS| > .2) verified by WGCNA and 886 DEPs in the control and withdrawal groups verified by differentially expressed analysis overlapped into 350 DEPs (Figure 5A). A PPI network was constructed containing 173 proteins (Figure 5B). To identify hub proteins in the PPI network, CytoHubba was used to analyze the 173 proteins with interactions by four algorithms. The intersection of the top 10 proteins of each algorithm was shown in the Venn diagram and seven hub proteins (Dlg3, Dlg4, Shank3, Grin2b, Camk2b, Camk2a and Syngap1) were obtained (Figure 5C), closely implicated with glutamatergic synapses and the calcium signaling pathway. This was confirmed by enrichment analysis of WGCNA mentioned above. All hub proteins were downregulated in the withdrawal group compared with control group, and upregulated in the diazepam group compared with withdrawal group. Based on the identified miRNA–mRNA pairs, we constructed an interaction network containing 49 miRNA–mRNA pairs, 31 miRNA and seven mRNA, visualized by Cytoscape (Figure 5D). By comparing seven hub mRNAs, Grin2b was found to be a potential target of 19 miRNAs. Camk2a and Dlg3 were the potential targets of 11 and seven miRNAs, respectively. Regarding miRNA targeting of these hub mRNAs, mmu-miR-491-5p were the main regulatory candidates based on the most interactions (degree = 6), indicating that it could be a biomarker of alcohol withdrawal.

### 3.7 Identification of hub proteins related to diazepam treatment

**FIGURE 6:**
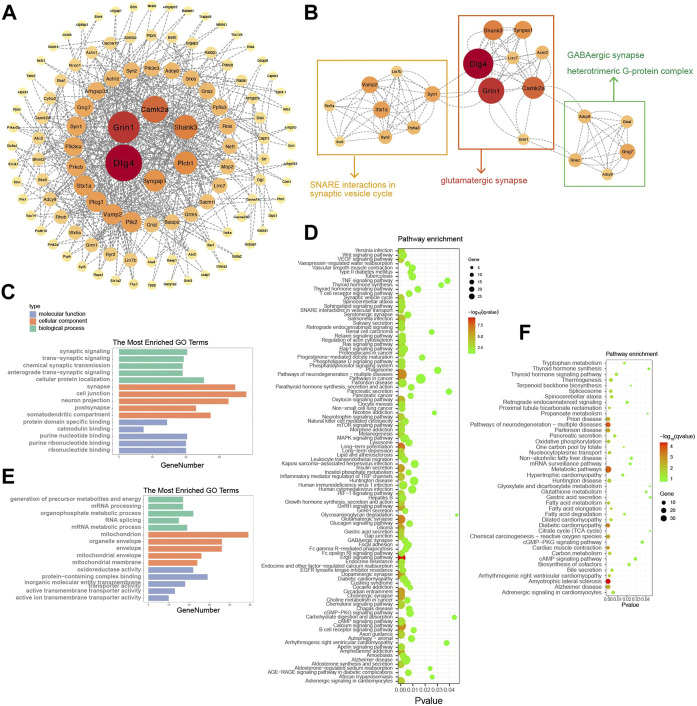
Identification of hub proteins related to diazepam treatment. **(A)** PPI network construction of 365 DEPs related to diazepam treatment. **(B)** The most significant MCODE cluster with 21 nodes and 114 edges. **(C)** Top 15 GO functional annotations of upregulated DEPs in Diazepam Group compared with Withdrawal Group. **(D)** KEGG pathway enrichment analysis of upregulated DEPs in Diazepam Group compared with Withdrawal Group. **(E)** Top 15 GO functional annotations of downregulated DEPs in Diazepam Group compared with Withdrawal Group. **(F)** KEGG pathway enrichment analysis of downregulated DEPs in Diazepam Group compared with Withdrawal Group.

To research the mechanism of action of diazepam on alcohol consumption and withdrawal, 365 diazepam-treatment-related DEPs were imported into the STRING online database to construct the PPI network (Figure 6A). An interaction network of 112 proteins was acquired. To identify hub proteins in the network, MCODE was applied for clustering proteins and the most significant MCODE cluster with 21 nodes and 114 edges is shown in Figure 6B. The related pathway involved glutamatergic synapses, SNARE interactions in the synaptic vesicle cycle, GABAergic synapses, and heterotrimeric G-protein complexes.

To further investigate the functions and mechanisms of diazepam-related proteins, GO and KEGG analyses were performed. In upregulated DEPs, the enriched GO annotations included cellular protein localization in the BP category, cell junction in the CC category, and purine nucleotide binding in the MF category. KEGG pathways included ErbB signaling pathway, long-term potentiation, glutamatergic synapses, pathways of neurodegeneration—multiple diseases, calcium signaling pathway, and dopaminergic synapses (Figures 6C, D). For downregulated DEPs, the enriched GO annotations included organophosphate metabolic process in the BP category, mitochondrion in the CC category, and protein-containing complex binding in the MF category. KEGG pathways included amyotrophic lateral sclerosis, pathways of neurodegeneration—multiple diseases, metabolic pathways, Parkinson’s disease, Alzheimer’s disease, fatty acid metabolism, oxidative phosphorylation, and Huntington’s disease (Figures 6E, F). We demonstrated that the mechanism of action of diazepam on AWS may involve upregulation of the ErbB signaling pathway, glutamatergic synapses, calcium signaling pathway, downregulated metabolic pathways, and oxidative phosphorylation. However, this needs experimental verification.

## 4 Discussion

To our knowledge, this is the first study to apply proteomics to explore potential biomarkers of diazepam treatment of AWS in mice. This large-scale assessment of protein expression changes contributes to our understanding of the effects of diazepam on AWS and the potential physiological and pharmacological actions. Here, we applied a label-free and iBAQ LC-MS/MS-based proteomics approach to identify seven hub proteins related to withdrawal (Dlg3, Syngap1, Grin2b, Dlg4, Camk2b, Shank3 and Camk2a) and significant alteration of a pathway most closely related to diazepam treatment of AWS, the glutamatergic synapse. Additionally, the calcium signaling pathway was demonstrated as a suitable biological correlate.

Excitatory synapses are most often localized on dendritic spines, characterized by an electron-dense matrix of receptors and supporting proteins collectively known as the postsynaptic density (PSD). This complex assembly of hundreds of distinct proteins dynamically changes its structure and composition during development and in response to synaptic activity. The PSD contains signaling molecules including the subunits of the glutamate receptors N-methyl D-aspartate (NMDA) receptor, alpha-amino-3-hydroxy-5-methyl-4-isoxazole propionate (AMPA) receptor, calcium/calmodulin dependent protein kinase II (CaMKII) and synGAP. Other prominent PSD proteins are scaffold molecules, including the PSD-95 family and Shank (Spiga et al., 2014).

Several studies have reported that chronic ethanol exposure followed by withdrawal induced excitotoxicity because the balance was destroyed between neuroadaptive changes (such as increased extracellular glutamate increased average amplitude (Gerace et al., 2021) and basal frequency (Zuo et al., 2019) of spontaneous excitatory postsynaptic currents; sEPSCs) in the duration of chronic ethanol exposure and the central nervous system inhibitory effect of ethanol. In early withdrawal (0–12 h), the inhibitory effect of ethanol was not exerted and excitatory synapses were in a state of overpotentiation, causing increased extracellular glutamate (Rossetti and Carboni, 1995), intense Ca^2+^ loading, p38 mitogen-activated protein kinase (MAPK) activation and oxidative stress, culminating in ATP depletion, mitochondrial injury (Jaatinen et al., 2003) and neuronal death (Skrzypiec et al., 2009; Jung and Mallet, 2018). However, with the extension of the withdrawal process, the levels of neurotransmitters often returned to normal, which was related to the changes in protein expression. Our results found two protective mechanisms that attenuated withdrawal-induced excitotoxicity. 1) The decreased expression of postsynaptic glutamate receptor GluN2B, mGlu7, and PSD proteins PSD-95, SAP102, Shank3 and CaMKII reduced synaptic potentiation, alleviating abnormal glutamatergic transmission. 2) Upregulation of glutamate transporter EAAT4 decreased extracellular glutamate concentration by taking up more glutamate into the glia.

Several studies have demonstrated that glutamatergic transmission was implicated with chronic ethanol exposure and withdrawal (Gerace et al., 2016; Gerace et al., 2018). For example, in mice with ethanol withdrawal, there were significant decreases in sEPSC amplitude and current kinetics, suggesting a decrease in postsynaptic glutamate transmission (Patel et al., 2022). This was similar to our proteomic analysis that showed that glutamatergic synapse signaling was decreased. NMDA receptor antagonist dizocilpine reduced both the physical signs of withdrawal and glutamate output (Rossetti and Carboni, 1995). We found that GluN2B was significantly downregulated (logFC = −14.40, *p* = .005) and AMPA subunit 4 (GluA4) was mildly upregulated (logFC = 1.60, *p* = .009) in mice in the withdrawal group compared with control group. NMDA amplitude was significantly decreased and the AMPA/NMDA ratio was significantly increased, indicating a selective decrease in postsynaptic glutamate transmission in the BLA-mPFCCRF1+ pathway in mice with alcohol withdrawal (Patel et al., 2022). Despite a previous report showing that ethanol withdrawal induced increased expression of metabotropic glutamate subtype 5 (mGlu5) receptor (Gerace et al., 2018), we found no significant difference for mGlu5. The expression levels of mGlu4 and mGlu7 had opposite trends (logFC = 15.24, *p* = .005 and logFC = −13.83, *p* = .005, respectively).

The PSD-95 family of proteins, known as synaptic membrane-associated guanylate kinases, are highly expressed at excitatory synapses (Won et al., 2017) and as scaffolding proteins, regulate clustering and function of NMDA receptors (Alele and Devaud, 2005), including PSD-95, PSD-93 and SAP102 (Chen et al., 2021). Several studies have demonstrated the relationship between PSD-95 and alcohol consumption. For example, alcohol consumption reduced the expression of PSD-95 in the dorsal hippocampus of rats (Marcolin et al., 2020). A reduction in PSD-95 expression could indicate a deficit in assembling clusters of glutamatergic receptors in postsynaptic membranes, making it difficult to respond to glutamatergic stimuli coming from other regions (Chen et al., 2011). We also found that PSD-95 (Dlg4), SAP102 (Dlg3) and Shank3 were downregulated (logFC = −1.42, *p* = .009; logFC = −1.28, *p* = .009; logFC = −6.66, *p* = .009, respectively) in the withdrawal group compared with control group. This suggests that the expression deficit of PSD-95 family and scaffolding proteins is a potential mechanism of ethanol-withdrawal-induced decreased postsynaptic glutamate transmission.

The extracellular glutamate concentration is tightly controlled by excitatory amino acid transporters (EAATs) (Brolese et al., 2015). Several studies have assessed the effects of chronic ethanol exposure on EAATs. For example, in a *Xenopus* oocyte expression system, decreased EAAT4 (Yoo et al., 2008) and EAAT3 (Kim et al., 2005) activity was observed in chronic ethanol exposure. However, some studies have demonstrated that chronic ethanol exposure increased EAAT expression. EAAT3 (EAAC1) showed significantly higher expression in the cerebral cortex and hippocampus in ethanol-withdrawn female rats (Alele and Devaud, 2005). Pharmacoproteomic results have demonstrated that ethanol exposure increased EAAT2 expression (Germany et al., 2018). The present study showed that ethanol withdrawal increased EAAT4 (Slc1a6) expression, which was the largest change among DEPs compared with the controls (logFC = 22.49, *p* = .005), suggesting uptake of more extracellular glutamate into the glia.

CaMKII was one of the PSD members in excitatory synapses, regulating NMDA-receptor-dependent synaptic potentiation. NMDA-receptor-mediated EPSCs mediate calcium flux into the postsynaptic compartment, primarily activating downstream CaMKII, resulting in autophosphorylation of the kinase, leading to induction and maintenance of synaptic potentiation that are crucial for neuronal development, synaptic and structural plasticity, learning, and memory (Lisman, 2017; Yong et al., 2021). Recently, a gene mutant mouse study identified that αCaMKII autophosphorylation-dependent remodeling of glutamatergic synapses is a plausible mechanism for behavior related to alcohol addiction (Mijakowska et al., 2017). Several studies have demonstrated reduced CaMKII expression (Ayers-Ringler et al., 2016) or activity (Christian et al., 2012) during ethanol withdrawal. For example, Thr286 dephosphorylation along with Thr305/306 phosphorylation shifted CaMKII kinase to an inactive state during alcohol withdrawal (Christian et al., 2012). The enhancement of sEPSCs and firing was blocked by a CaMKII inhibitor in ethanol-withdrawn rats, reducing ethanol intake (Zuo et al., 2019). However, some research has shown that abstinence from alcohol exposure induced an undercurrent of CaMKII kinase activity, which may have promoted aberrant glutamatergic responses (Natividad et al., 2018). Our results showed that decreased expression levels of CaMKIIα and CaMKIIβ (logFC = −2.75, *p* = .009; logFC = −1.02, *p* = .016, respectively) in ethanol-withdrawn mice. We propose that reduced CaMKII expression or activity may be considered as a protective mechanism to resist abnormal synaptic potentiation during ethanol deprivation.

SynGAP is a neuron-specific Ras and Rap GTPase-activating protein with high expression in the PSD fraction (WGt et al., 2015) of excitatory neurons and phosphorylated by CaMKII (Oh et al., 2004) to regulate neural development, synaptic plasticity (Zhang et al., 2020), and the trafficking of glutamate receptors (Carlisle et al., 2008). Mutations in the *SynGAP1* gene have been linked to stroke (Zhang et al., 2020; Yang et al., 2022) and neurodevelopmental disorders, such as cognitive dysfunction (Lai et al., 2021; Kilinc et al., 2022), autism spectrum disorders (Harris et al., 2021), schizophrenia (Gamache et al., 2020) and epilepsy (Creson et al., 2019). However, the relationship between SynGAP and ethanol consumption has not been reported so far. Our results found that SynGAP1 was significantly downregulated in ethanol-withdrawn mice (logFC = −3.47, *p* = .009), which may be explained by the proposal that GluN2B-containing NMDA receptors and CaMKII act upstream of SynGAP (Wang et al., 2013). SynGAP is considered to be a negative regulator of Ras (Yang et al., 2022). Phosphorylation of synGAP by CaMKII increased its Ras GTPase-activating activity (Oh et al., 2004), preventing the activation of Ras and potentiating p38 MAPK signaling (Rumbaugh et al., 2006). SynGAP dissociation from the MUPP1–CaMKII complex resulted in its dephosphorylation, accompanied by p38 MAPK inactivation (Krapivinsky et al., 2004), which was consistent with our KEGG analysis by WGCNA. The clustered genes of the MAPK signaling pathway (GeneRatio = 21/330, p.adjust = .009) was from turquoise modules (downregulated, withdrawal related), but not from blue modules (upregulated, withdrawal related). In slices from rats subjected to 1-day withdrawal from CIE treatment, the reduction in MAPK phosphorylation during post-tetanic potentiation was observed (Roberto et al., 2003). However, some researchers have reported that p38 MAPK was activated by ethanol withdrawal from chronic ethanol exposure (Jung et al., 2016; Tian et al., 2016; Ryou et al., 2017). These apparently contradictory results could be because of the differences in the species, sample type, model system, and timing of exposure and withdrawal. In summary, our results showed that in the later period of ethanol withdrawal, a series of neuroadaptive changes occurred to attenuate withdrawal-induced excitotoxicity (i.e., increased extracellular glutamate).

In the miRNA-mRNA interaction analysis, mmu-miR-491-5p were the main regulatory candidates and could be a biomarker of AUD. As known, MicroRNA-491-5p (miR-491-5p) plays an important role in regulating cell proliferation and migration (Liu et al., 2020). Besides, mmu-miR-491-5p also involved neurodevelopment and angiogenesis, for example, Tang W revealed that miR-491-5p downregulation alleviated neurological dysfunction, promoted the recovery of regional cerebral blood flow, increased the number of lectin-stained microvessels, and increased the survival of neurons after traumatic brain injury (Tang et al., 2022). However, we need more experiments to confirm the relationship between miR-491-5p and AUD such as detection in the serum of patients with AUD.

Diazepam is a well-known psychoactive drug widely used worldwide for the treatment of anxiety, seizures, alcohol withdrawal syndrome, muscle spasms, sleeplessness, and agitation. It is the number of the benzodiazepine family, substances known to primarily act by binding and enhancing GABA(A) receptors (Markin et al., 2021). Several studies have reported that diazepam suppressed ethanol withdrawal symptoms and canceled out the working memory impairments and glucocorticoid alterations in the alcohol-withdrawn animals (Mhatre et al., 2001; Dominguez et al., 2018). Alcohol is an agonist of the GABAA receptor (Lovinger and Homanics, 2007). However, chronic ethanol exposure and withdrawal induce GABAA receptor adaptive change. The study has shown that the maximal density of GABAA receptor-mediated current was reduced significantly by 33 or 28% after chronic ethanol treatment or ethanol withdrawal, respectively (Sanna et al., 2003). Accordantly, our results demonstrated that the proteins of Turquoise modules were clustered to GABAergic synapse (GeneRatio = 19/330, p.adjust <.001), not Blue modules, suggesting downregulated GABAergic synapse in withdrawal group compared with control group. Meanwhile, GABAergic synapse of diazepam group was upregulated compared with withdrawal group (GeneRatio = 6/147, p.adjust = .013), indicating diazepam activated GABAA receptor. In addition, our research found that glutamatergic synapse of diazepam group was upregulated compared with withdrawal group (GeneRatio = 14/147, p.adjust = 3.07E-07), probably resulting from that CNS inhibition induced by diazepam excited GABA receptor needs to be balanced by upregulated glutamatergic synapse. However, the potential mechanism needs further research to clarify.

Prior research has shown that diazepam increased aspartate concentration (Markin et al., 2021). Aspartic acid is an agonist of the excitatory AMPA and NMDA receptors. In the present study, we showed that NMDA receptors (Grin1) and metabotropic glutamate receptors (MGlu3, MGlu5 and MGlu7) were significantly upregulated in the diazepam group compared with the withdrawal group. Therefore, it is conceivable that diazepam-induced elevated aspartic acid levels may alleviate withdrawal-induced glutamatergic synapse inhibition. The physiological reasons for this deserve further investigation.

## 5 Conclusion

We used a label-free iBAQ proteomics approach and bioinformatic analysis to determine protein expression profiles of the brain and identify the hub proteins that are associated with ethanol withdrawal or diazepam treatment (i.e., Dlg3, Dlg4, Shank3, Grin2b, Camk2b, Camk2a and Syngap1). In enrichment analysis, glutamatergic synapse was the most significant pathway related to AUD that may be a potential molecular target for new interventional strategies. The pharmacological mechanism of action of diazepam in the treatment of AWS may involve increasing aspartate concentrations, contributing to alleviating withdrawal-induced glutamatergic synapse inhibition. Continued investigation of the detailed roles of the proteins may help gain insight into the mechanisms responsible for the development of AUD that may eventually lead to the discovery of novel diagnostic markers and therapeutic targets.

## Data Availability

The original contributions presented in the study are included in the article/Supplementary Materials, further inquiries can be directed to the corresponding authors.
