# Supplementary figures and images for: Proteomics and weighted gene correlated network analysis reveal glutamatergic synapse signaling in diazepam treatment of alcohol withdrawal
Source: Front Pharmacol. 2023 Jan 11;13:1111758. doi: 10.3389/fphar.2022.1111758 (PMC9873974; doi:10.3389/fphar.2022.1111758)

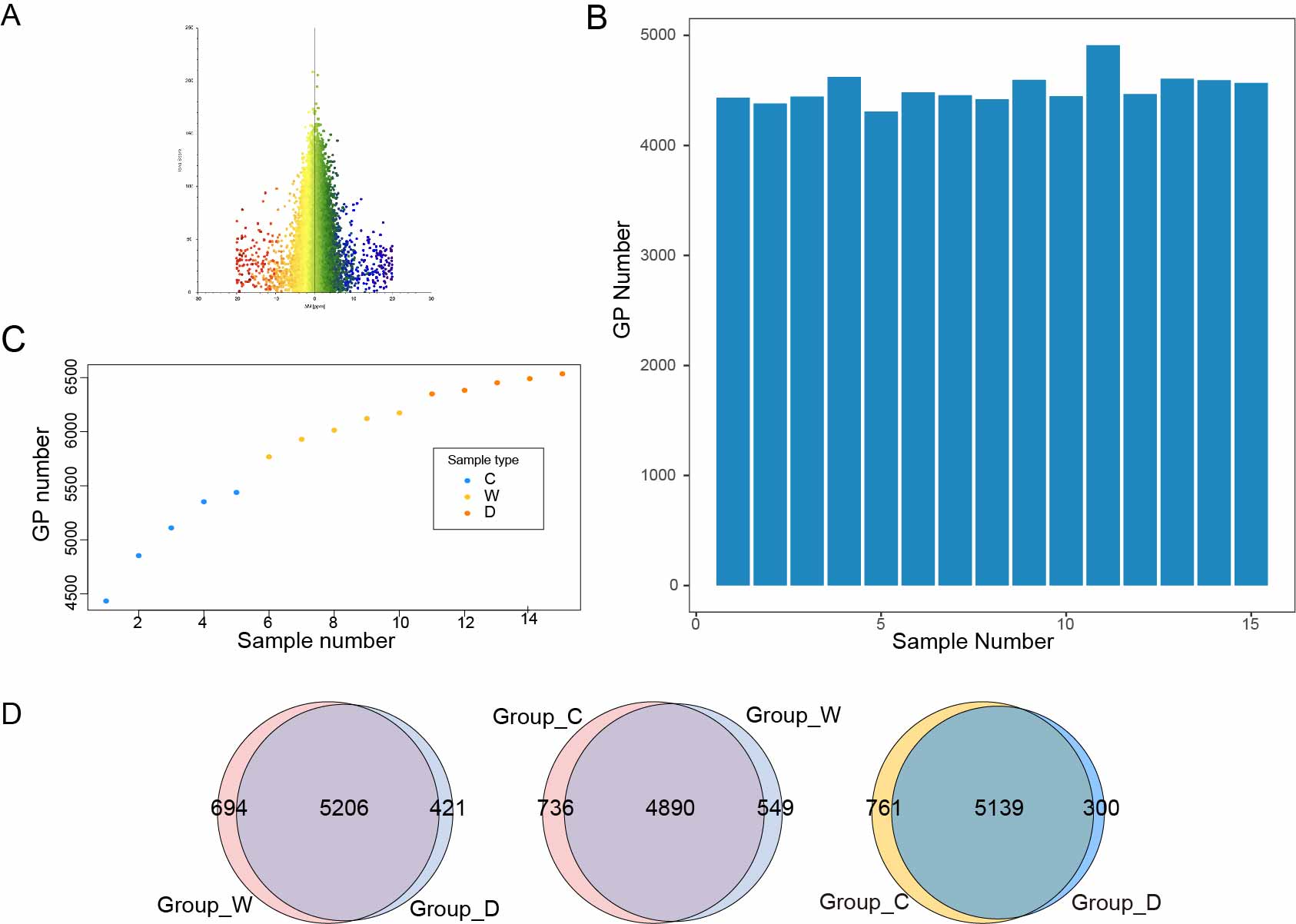

Supplement: Supplementary file 1 [file Image1.JPEG]

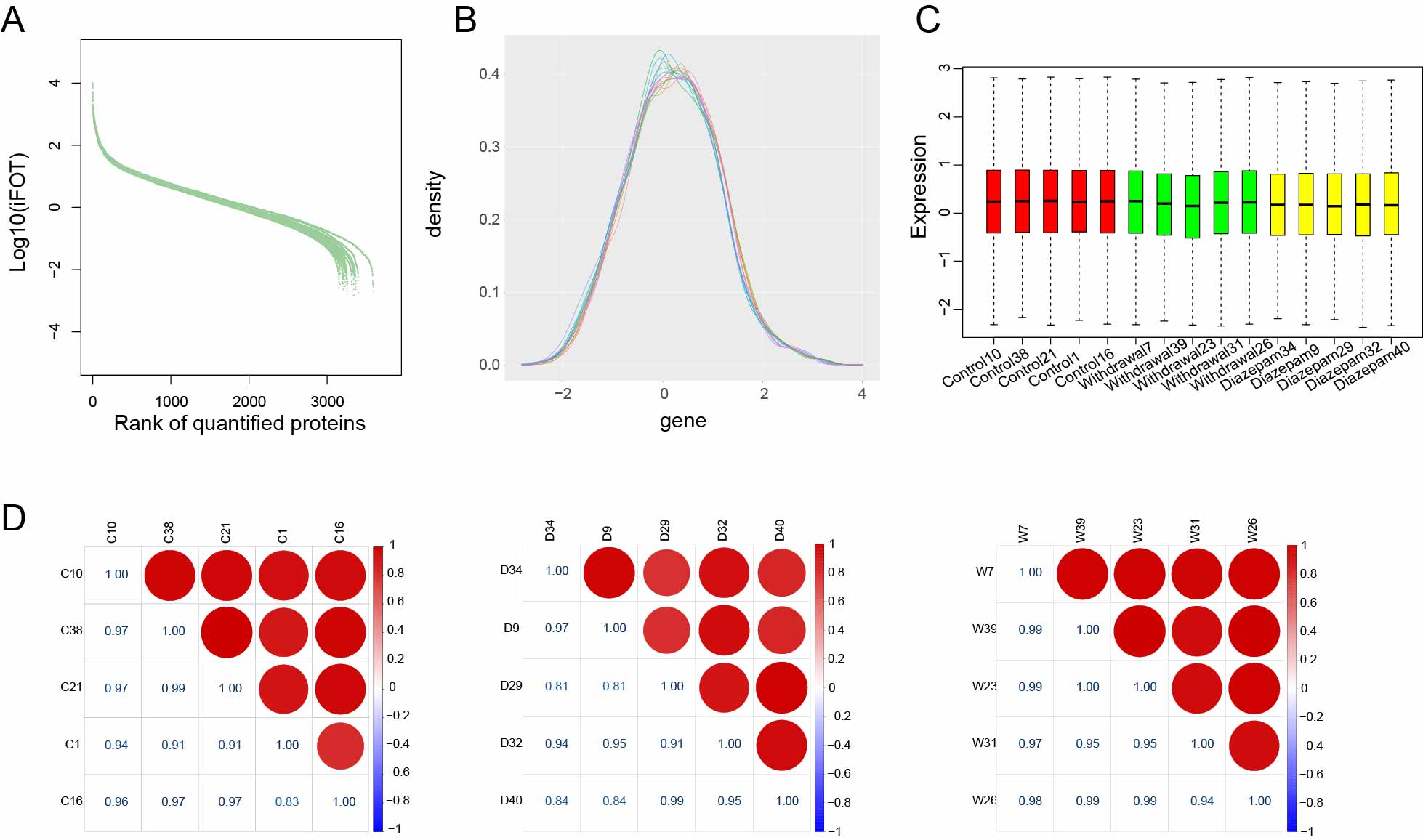

Supplement: Supplementary file 2 [file Image2.JPEG]
